# Human Umbilical Cord Mesenchymal Stem Cells Ameliorate Hepatic Stellate Cell Activation and Liver Fibrosis by Upregulating MicroRNA-455-3p through Suppression of p21-Activated Kinase-2

**DOI:** 10.1155/2021/6685605

**Published:** 2021-02-25

**Authors:** Qing Zhou, Tengfei Gu, Yong Zhang, Hongda Li, Xuemei Zhuansun, Sanrong Xu, Yuting Kuang

**Affiliations:** ^1^Department of General Surgery, The First Affiliated Hospital of Soochow University, Suzhou 215006, China; ^2^Department of General Surgery, Affiliated Hospital of Jiangsu University, Zhenjiang 212001, China; ^3^Department of Anesthesiology, People's Hospital of Lianshui County, Jiangsu Province, Lianshui 223400, China; ^4^Laboratory Animal Research Center, Medical College of Soochow University, Suzhou 215123, China

## Abstract

Mesenchymal stem cells (MSCs) were shown to have potential therapeutic effects for treatment of liver fibrosis, and dysregulated expression of microRNAs (miRNAs) played a pivotal role in the pathogenesis of liver fibrosis by regulating their downstream target genes. However, the mechanism by which MSCs affect the progression of liver fibrosis by regulating miRNA expression remains unclear. Here, we investigated whether human umbilical cord MSCs (HUC-MSCs) attenuated hepatic fibrosis by regulating miR-455-3p and its target gene. Significantly upregulated miRNA (miR-455-3p) was screened out by GEO datasets analysis and coculture HUC-MSCs with hepatic stellate cell (HSC) LX-2 cells. p21-activated kinase-2 (PAK2) was forecasted to be the target gene of miR-455-3p by bioinformatics analyses and confirmed by luciferase reporter assay. HUC-MSCs were transplanted into mice with carbon tetrachloride- (CCl_4_-) induced liver fibrosis, the result showed that HUC-MSC transplantation significantly ameliorated the severity of CCl_4_-induced liver fibrosis, attenuated collagen deposition, improved liver function by reducing the expression of alanine aminotransferase (ALT) and aspartate aminotransferase (AST) in serum, upregulated miR-455-3p, and suppressed PAK2 expression of liver tissue in mice. Taken together, our study suggests that HUC-MSCs inhibit the activation of HSCs and mouse CCl_4_-induced liver fibrosis by upregulation of miR-455-3p through targeting PAK2.

## 1. Introduction

Liver fibrosis and cirrhosis are common pathological processes of many chronic liver diseases, including chronic hepatitis B and C, alcoholic steatohepatitis, nonalcoholic steatohepatitis, and autoimmune hepatitis, which can eventually lead to liver failure or liver cancer. Hepatic stellate cells (HSCs) are widely accepted to be the fibrogenic cell type in the liver [1]. The characteristic of fibrosis is accompanied by the activation and proliferation of HSCs and increased production of excessive extracellular matrix [2, 3]. Currently, there are still few strategies to reverse liver fibrosis except orthotopic liver transplantation, but organ donor shortage, complications of surgery, and immunosuppression limit its application [4]. Therefore, there is an urgent need to find a new alternative treatment strategy.

Mesenchymal stem cells (MSCs) are multipotent stem cells, which can be isolated from bone marrow, adipose tissue, umbilical cord, and other tissues [5, 6]. There have been demonstrated that MSCs have an inflammatory tendency and can homing to the injured site to play an anti-inflammatory role [7, 8]. Recently, accumulating evidences suggest that exogenous MSC treatment could ameliorate HSC activation and liver fibrosis, differentiate into hepatic cells, and promote the recovery of liver function [9, 10].

MicroRNAs (miRNAs), a class of endogenous noncoding RNAs, approximately 22 nucleotides long, function as negatively regulate protein translation by binding to the 3′ untranslated region (3′ UTR) of mRNAs of their target gene [11]. Recent studies have shown that miRNAs regulate the activation of HSCs and the progress and development of liver fibrosis. For example, miR-125b promotes activation of HSCs and liver fibrosis by activating the ras homolog gene family, member A (RhoA) signaling [12], miR-26b-5p inhibits liver fibrogenesis by targeting platelet derived growth factor receptor-*β* (PDGFR-*β*) [13], and miR-455-3p alleviates HSC activation and prevents liver fibrosis by suppressing heat shock factor 1 (HSF1) expression [14]. However, the underlying mechanisms of MSCs alleviating HSC activation by regulating miRNA expression remains unknown.

In this study, we used two miRNA profile data (GSE33857 and GSE77271) about liver fibrosis which were downloaded from publicly available GEO datasets and screened out five common differentially expressed miRNAs (DEMs) (miR-342-3p, miR-125a-5p, miR-455-3p, miR-574-5p, and miR-193a-3p) which were upregulated or downregulated in experimental animals or human liver fibrosis samples. The result was verified by quantitative real-time reverse transcription-polymerase chain reaction (qRT-PCR) in activated HSCs induced by transforming growth factor *β*1 (TGF*β*1). Subsequently, we cocultured activated HSCs with human umbilical cord mesenchymal stem cells (HUC-MSCs) and found that miR-455-3p expression was the most significant difference compared with the HSC group cultured alone. These findings were subsequently verified in mouse models of liver fibrosis treated with HUC-MSCs. Finally, our results demonstrated that HUC-MSC treatment ameliorated liver fibrosis by upregulating miR-455-3p through suppression of p21-activated kinase-2 (PAK2).

## 2. Materials and Methods

### 2.1. Isolation, Culture, and Expansion of HUC-MSCs

HUC-MSCs were isolated as described previously [15]. Umbilical cords were donated by mothers who provided written informed consent for research purposes. The collection and isolation procedure were both approved by the Ethics Committee of Soochow University (Suzhou, China). Cells were cultured in Dulbecco's modified Eagle's medium (DMEM) supplemented with 10% fetal bovine serum (FBS) (Gibco, Grand Island, NY, USA), 1 mmol/L GlutaMAX™ (Gibco), and 100 IU/mL penicillin/streptomycin (Gibco) at 37°C in a 5% CO_2_-humidified atmosphere with medium changes every 2-3 days and were subcultured by using 0.25% trypsin-EDTA (Thermo Fisher Scientific, Waltham, MA, USA) upon reaching 80%-90% confluence. The ability to differentiate into osteoblasts and adipocytes and the surface antigen profile of HUC-MSCs were confirmed as previously reported [15, 16].

### 2.2. HSC Culture and Treatment

The human HSC line LX-2 cells were purchased from the Cell Center of Shanghai Institutes for Biological Sciences (Shanghai, China). Cells were cultured in DMEM supplemented with 10% FBS, GlutaMAX™, and 100 IU/mL penicillin/streptomycin. For activation, LX-2 cells at 70-80% confluence were starved for 24 hours in DMEM medium containing 0.2% FBS and then treated with TGF*β*1 (Sigma, St. Louis, MO, USA) at a concentration of 10 ng/mL for 48 hours.

### 2.3. HUC-MSCs/LX-2 Cocultures

Fully activated LX-2 cells were cultured alone or cocultured with HUC-MSCs using transwell inserts (24 mm diameter, 0.4-*μ*m pore size, Corning, NY, USA) to assess the effects of HUC-MSCs on LX-2 cells. All coculture experiments were performed with LX-2 cells in the lower chamber and HUC-MSCs in the upper compartment using DMEM medium with 10% FBS. LX-2 cells were collected after culturing for 48 hours.

### 2.4. Cell Proliferation Assays

The proliferation of LX-2 cells was measured using a Cell Counting Kit-8 (CCK8) assay (Dojindo Molecular Technologies, Inc., Kumamoto, Japan). In brief, LX-2 cells were seeded in the lower chamber of 6-well plate using transwell inserts at a density of 5 × 10^4^ cells per well, while in the coculture group the same number of HUC-MSCs was seeded in the upper compartment. After culturing for 48 hours, the upper department was removed, and the CCK8 reagent was added to the lower chamber containing LX-2 cells and incubated for additional 2 hours according to the manufacturer's protocol. Optical densities were measured using a microplate reader (SpectraMax M5; Molecular Devices, Sunnyvale, CA, USA) at a wavelength of 450 nm.

### 2.5. miR-455-3p Transfection

miR-455-3p mimics, inhibitors, and their negative controls (NC) were purchased from GenePharma (Shanghai, China). LX-2 cells were transfected with Lipofectamine™ 3000 reagent (Invitrogen, Carlsbad, CA, USA) according to the manufacturer's instructions. Then, the cells were maintained at 37°C in a humidified atmosphere containing 5% CO_2_ for 48 hours.

### 2.6. Luciferase Reporter Assay

The 3′ UTR sequence of human PAK2 that contained the putative wild-type (WT) or mutant (MUT) miR-455-3p binding site was inserted into the pGL3 luciferase vector (Promega, Madison, WI, USA). For the luciferase reporter experiments, LX-2 cells were cotransfected with the above constructed 3′ UTR luciferase vector and miR-455-3p mimics or NC using Lipofectamine™ 3000 reagent. Cells were harvested 48 hours after transfection for dual-luciferase reporter assay by a fluorescence detector (Promega). The relative firefly luciferase activity was normalized to Renilla luciferase activity.

### 2.7. Animal Models

Animal experiments were performed according to the guidelines of Institutional Animal Care and Use Committee of Soochow University (Suzhou, China). Male C57BL/6 mice of 8-10 weeks age were purchased from Shanghai SLAC Laboratory Animal Co., Ltd. (Shanghai, China). Liver fibrosis mice were induced by carbon tetrachloride (CCl_4_) (20% in olive oil, 5 *μ*L/g) intraperitoneal administration twice a week for 12 weeks, while mice in the control group were treated with olive oil. Each experimental group contained 6 mice. Mice were divided into three groups: control group, CCl_4_ group, and CCl_4_+HUC-MSC group. CCl_4_+HUC-MSC group mice were injected 5 × 10^5^ HUC-MSCs in 0.3 mL phosphate-buffered saline (PBS) by the tail vein once a week at the 6th week after CCl_4_ administration for six times, while mice in the control and CCl_4_ groups were injected with only 0.3 mL PBS during the same period. At the end of the 12th week, venous blood was obtained from the retroorbital vein to measure liver serum markers. Then, all mice were sacrificed and liver lobes were obtained for further analyses.

### 2.8. Histology

Liver tissues were fixed in 4% paraformaldehyde and embedded in paraffin. Sections were cut at 5 *μ*m thickness and stained with hematoxylin-eosin (H&E). Collagen deposition of sections was detected by Masson's trichrome staining and Sirius red staining using standard protocols.

### 2.9. Immunofluorescence and Immunohistochemistry

Immunofluorescence staining was performed to assess *α*-smooth muscle actin (*α*-SMA) and collagen 1*α*1 (Col1*α*1) on LX-2 cells as routine protocols. Briefly, LX-2 cells were seeded into 6-well plates and divided into three groups: LX-2 group, LX-2 cells cultured alone; LX-2+TGF*β*1 group, LX-2 cells cultured alone and treated with TGF*β*1 for activation; and LX-2+HUC-MSCs group, LX-2 cells treated with TGF*β*1 and then co-cultured with HUC-MSCs. After being cultured, the cells were washed twice with PBS and then fixed with 4% paraformaldehyde and permeabilized with 0.1% Triton X-100 in PBS for 20 minutes at room temperature. After being washed with PBS, the cells were incubated with 10% goat serum in PBS for blocking nonspecific binding sites and then incubated overnight with primary antibodies specific to *α*-SMA (1 : 200; Abcam, Cambridge, MA, USA) or Col1*α*1 (1 : 200; Abcam) at 4°C. After being washed with PBS, the cells were finally incubated with Dylight 649-conjugated or Dylight 488-conjugated secondary antibody for 2 hours in the dark. After being incubated with DAPI (Beyotime, Shanghai, China) for staining nuclei, the images of cells were acquired using a fluorescence microscope (OlympusBX-53, Tokyo, Japan).

For immunofluorescence staining and immunohistochemical analysis of liver tissues, paraffin-embedded tissue sections were dewaxed in xylene and dehydrated in a series of graded alcohols; heat-induced antigen retrieval was accomplished by microwaving in citric saline for 15 minutes. The sections were further blocked in PBS plus 0.025% Tween 20 with 2% bovine serum albumin. For immunofluorescence staining, the sections were incubated with primary antibodies specific for *α*-SMA (1 : 200; Abcam) and PAK2 (1 : 200; Abcam), then were further incubated with Dylight 649-conjugated and Dylight 488-conjugated secondary antibody; finally, the samples were incubated with DAPI (Beyotime). For immunohistochemical analysis, after blocking, the sections were incubated with primary antibodies specific for *α*-SMA (1 : 200; Abcam), Col1*α*1 (1 : 200; Abcam), and PAK2 (1 : 100; Abcam) overnight at 4°C. After washing with PBS, the sections were incubated with horseradish peroxidase- (HRP-) conjugated secondary antibody for 1 hour at room temperature. Subsequently, the signal was visualized by 3,3′-diaminobenzidine tetrachloride, and the nuclei were counterstained with hematoxylin.

### 2.10. Hepatic Hydroxyproline Content

The hepatic hydroxyproline level was measured using an assay kit (Nanjing Jiancheng Bioengineering Institute, Nanjing, China), according to the manufacturer's instructions.

### 2.11. Quantitative Real-Time PCR

Isolation of total RNA from cultured LX-2 cells or liver tissues was conducted using Trizol reagents (Invitrogen, Carlsbad, CA, USA). cDNA was synthesized using the PrimeScript RT reagent kit (Takara, Shanghai, China). qRT-PCR was performed in triplicate using the SYBR Premix Ex Taq (Takara) on the ABI ViiA 7Dx real-time PCR system (Life Technologies, NY, USA). The expression levels of mRNA of interest and microRNA were calculated according to the comparative threshold cycle value (2^−*ΔΔ*Ct^) method and were normalized against *β*-actin or U6. All of the primers were synthesized by GENEWIZ (Suzhou, China). The primer sequences used in this study were as follows: *α*-SMA (human), forward: 5′-CTATGAGGGCTATGCCTTGCC-3′, reverse: 5′-GCTCAGCAGTAGTAACGAAGGA-3′; Col1*α*1 (human), forward: 5′-GTGCGATGACGTGATCTGTGA-3′, reverse: 5′-CGGTGGTTTCTTGGTCGGT-3′; PAK2 (human), forward: 5′-CACCCGCAGTAGTGACAGAG-3′, reverse: 5′-GGGTCAATTACAGACCGTGTG-3′; *β*-actin (human), forward: 5′-AGAGCTACGAGCTGCCTGAC-3′, reverse: 5′-AGCACTGTGTTGGCGTACAG-3′; *α*-SMA (mouse), forward: 5′-GGCACCACTGAACCCTAAGG-3′, reverse: 5′-ACAATACCAGTTGTACGTCCAGA-3′; Col1*α*1 (mouse), forward: 5′-CTGGCGGTTCAGGTCCAAT-3′, reverse: 5′-TTCCAGGCAATCCACGAGC-3′; PAK2 (mouse), forward: 5′-AACGGAGAGCTAGAAGACAAGC-3′, reverse: 5′-TGGAACAGAAGGCAAAGGTTT-3′; *β*-actin (mouse), forward: 5′-GGCTGTATTCCCCTCCATCG-3′, reverse: 5′-CCAGTTGGTAACAATGCCATGT-3′; miR-455-3p, forward: 5′-GCGCAGTCCATGGGCAT-3′, reverse: 5′-CAGTGCGTGTCGTGGAGT-3′; and U6, forward: 5′-CTCGCTTCGGCAGCACATATACT-3′, reverse: 5′-ACGCTTCACGAATTTGCGTGTC-3′.

### 2.12. Western Blot Analysis

Cells and liver tissues were lysed in RIPA buffer (Millipore, Billerica, MA, USA), and total proteins were extracted. The protein concentration was measured using a BCA protein assay kit (Beyotime, Nanjing, China). Proteins used for Western blot analysis were performed routinely; membranes were incubated with primary antibodies against *α*-SMA (1 : 1000; Abcam), Col1*α*1 (1 : 2000; Abcam), GAPDH (1 : 2500; Abcam), and PAK2 (1 : 2000; Abcam) overnight at 4°C. Then, HRP-conjugated goat anti-rabbit IgG (1 : 1000; Cell Signaling Technology, Beverly, MA, USA) secondary antibody was employed at 37°C for 1 hour. The immunobands were visualized using the chemiluminescence HRP substrate (Millipore). The protein expression was standardized to GAPDH and quantified using Gel-Pro Analyzer software (Media Cybernetics, Rockville, MD, USA).

### 2.13. Statistical Analysis

The statistical analyses were performed by using GraphPad Prism 6 (GraphPad Software Inc., San Diego, CA, USA). Experimental data are expressed as the mean ± SD. Statistical significance was determined using the two-tailed Student's *t*-test for the comparison of two groups. For comparison among multiple groups, one-way analysis of variance (ANOVA) was performed. A value of *P* < 0.05 was considered statistically different.

## 3. Results

### 3.1. HUC-MSCs Inhibit Proliferation and Activation of HSCs

**Figure 1:**
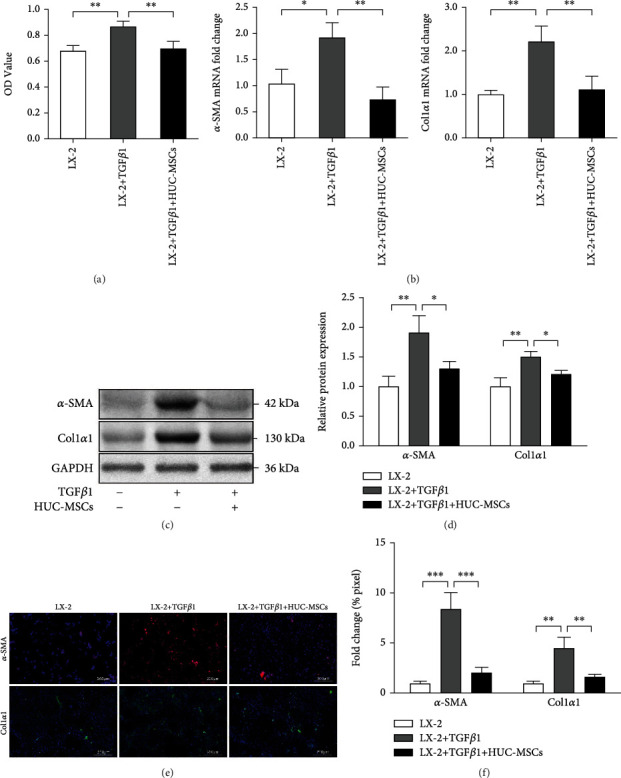
HUC-MSCs inhibit proliferation and activation of HSCs. (a) CCK8 assay analysis of HUC-MSCs on inhibition of TGF*β*1-mediated proliferation in LX-2 cells. (b) The mRNA levels of *α*-SMA and Col1*α*1 were measured by qRT-PCR. (c) The protein levels of *α*-SMA and Col1*α*1 were measured by Western blot. (d) Quantitation of *α*-SMA and Col1*α*1 levels from three independent Western blot analyses. (e) Immunofluorescence images of LX-2 cells, the red fluorescence represents *α*-SMA, and the green fluorescence represents Col1*α*1. Scale bars, 200 *μ*m. (f) Quantification of *α*-SMA and Col1*α*1 positive areas. Percentages of immunoreactive areas were expressed as relative values to those in the LX-2 group. The quantitative data are represented as the mean ± SD. Each experiment was repeated three times. ^∗^*P* < 0.05, ^∗∗^*P* < 0.01, and ^∗∗∗^*P* < 0.001.

To ascertain whether HUC-MSC transplantation affects the proliferation of HSCs, human HSC cell line LX-2 cells were cocultured with HUC-MSCs using a coculture transwell chamber. CCK-8 assays were performed to measure the cell proliferation of LX-2 cells. For activation, LX-2 cells were incubated with TGF*β*1. The results showed that TGF*β*1 treatment significantly promoted LX-2 cell proliferation, while HUC-MSCs notably reversed this effect (Figure 1(a)). To investigate whether HUC-MSCs could influence HSC activation, LX-2 cells were cultured in the absence of serum for starvation and activated with TGF*β*1. The expression of *α*-SMA and Col1*α*1 (two markers of activated HSCs) mRNA and protein in TGF*β*1 treatment group was highly increased, as determined by qRT-PCR, Western blot, and immunostaining analyses compared with LX-2 cultured in the absence of TGF*β*1, which indicated successful activation of LX-2 cells (Figures 1(b)–1(f)). Meanwhile, coculture with HUC-MSCs for 48 hours led to an obvious downregulation of *α*-SMA and Col1*α*1 mRNA expression, as determined by qRT-PCR (Figure 1(b)), and the inhibition effect of HUC-MSCs on HSC activation was in a dose-dependent manner (Supplementary Figure 1(a) and 1(b)). The expression of *α*-SMA and Col1*α*1 was also downregulated confirmed by repeated Western blot and immunostaining analysis, suggesting that TGF*β*1-mediated activation of LX-2 cells was inhibited by HUC-MSCs (Figures 1(c)–1(f)).

### 3.2. HUC-MSCs Promote Inactivation of LX-2 Cells by Upregulating miR-455-3p

**Figure 2:**
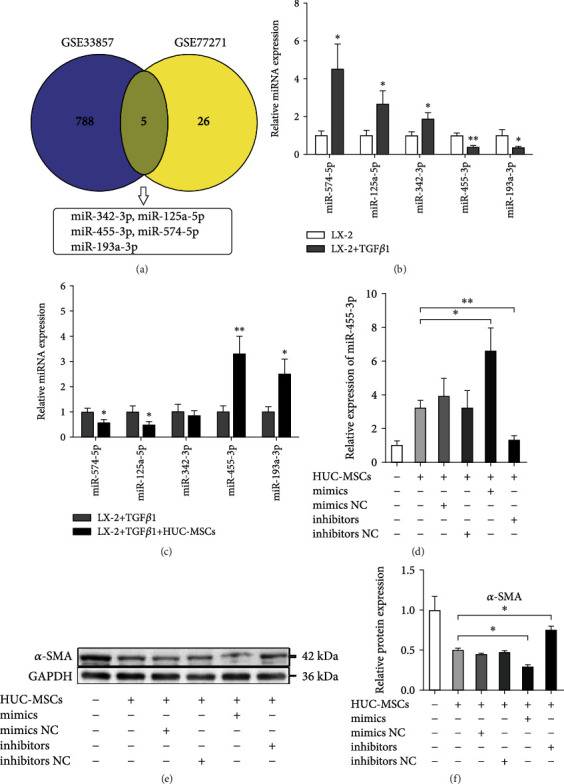
HUC-MSCs promote inactivation of LX-2 cells by upregulating miR-455-3p. (a) Venn diagram of DEMs about liver fibrosis from two miRNA profile data. (b) Comparison of qRT-PCR results of five selected DEMs in LX-2 cells treated with or without TGF*β*1. (c) Comparison of the expression levels of five selected DEMs in TGF*β*1-activated LX-2 cells cultured alone or cocultured with HUC-MSCs. (d) The expression levels of miR-455-3p were examined in TGF*β*1-treated LX-2 cells cocultured with HUC-MSCs and then were transfected with miR-455-3p mimics, mimics NC, inhibitors, or inhibitors NC, respectively. (e) The protein levels of *α*-SMA measured by Western blot in each group. (f) Quantitation of *α*-SMA levels from three independent Western blot analyses. The quantitative data are represented as the mean ± SD. Each experiment was repeated three times. ^∗^*P* < 0.05 and ^∗∗^*P* < 0.01.

Increasing evidence demonstrated that miRNAs regulate a wide range of cellular processes, including cell proliferation, differentiation, and apoptosis. In addition, aberrant miRNA expression may associate with the development of multiple diseases, including liver fibrosis. Here, we screened out five common differentially expressed miRNAs (DEMs) (miR-342-3p, miR-125a-5p, miR-455-3p, miR-574-5p, and miR-193a-3p) about liver fibrosis from two miRNA profile data (GSE33857 and GSE77271) which were downloaded from publicly available GEO datasets (Figure 2(a)). Then, the result was verified by qRT-PCR in activated HSC LX-2 cells induced by TGF*β*1 (Figure 2(b)). Subsequently, we cocultured activated LX-2 cells with HUC-MSCs and found that miR-455-3p expression was the most significant difference in the five DEMs compared with LX-2 cells cultured alone, and it indicated that HUC-MSCs significantly upregulated miR-455-3p expression in TGF*β*1-activated LX-2 cells (Figure 2(c)). Therefore, we chose miR-455-3p for a further study. To validate whether HUC-MSCs inhibit LX-2 cell activation by upregulating miR-455-3p, TGF*β*1-activated LX-2 cells were cocultured with HUC-MSCs for 48 hours and were transfected with miR-455-3p mimics, mimics NC, inhibitors, or inhibitors NC, respectively. We first confirmed the upregulation of miR-455-3p upon transfection of LX-2 with miR-455-3p mimics or downregulation of miR-455-3p upon transfection with miR-455-3p inhibitors (Figure 2(d)), and we observed that reduced fibrosis marker *α*-SMA levels upon miR-455-3p mimic transfection and increased *α*-SMA levels upon miR-455-3p inhibitor transfection (Figures 2(e) and 2(f)). The results showed that upregulation of miR-455-3p expression could improve the inactivation of LX-2 cells induced by HUC-MSCs, while downregulation of miR-455-3p expression could reverse the inhibitory effect. These observations indicated that HUC-MSCs suppressed the activity of LX-2 cells by upregulating miR-455-3p.

### 3.3. miR-455-3p Regulates PAK2 Expression by Directly Targeting the 3′ UTR of Its mRNA

**Figure 3:**
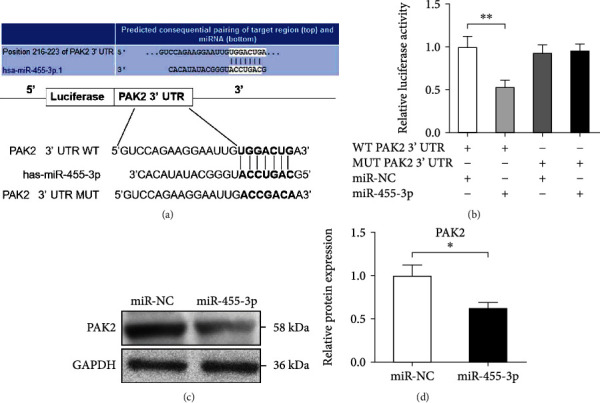
miR-455-3p regulates PAK2 expression by directly targeting the 3′ UTR of its mRNA. (a) Predicted miR-455-3p targeting sequence in 3′ UTR of WT or MUT PAK2 mRNA. (b) Dual-luciferase reporter assay of LX-2 cells cotransfected with WT or MUT PAK2 3' UTR reporter and miR-455-3p mimics or mimics NC. (c) PAK2 protein expression analysis in LX-2 cells transfected with miR-455-3p mimics using Western blotting. (d) Quantitation of PAK2 protein levels from three independent Western blot analyses. The quantitative data are represented as the mean ± SD. Each experiment was repeated three times. ^∗^*P* < 0.05 and ^∗∗^*P* < 0.01.

We then investigated the underlying mechanism by which miR-455-3p regulates HSC activation and to identify the downstream target genes of miR-455-3p, and we performed bioinformatics analyses using Microt4, miRanda, miRDB, RNA22, and TargetScan databases and focused on PAK2, an activated receptor of TGF*β*. The 3′ UTR of PAK2 mRNA contained a complementary site for the seed region of miR-455-3p (Figure 3(a)). Moreover, PAK2 has been reported to be a downstream target gene of miR-455-3p in human osteoblasts and in human colon adenocarcinoma cells [17, 18]. However, whether miR-455-3p regulates PAK2 expression in HSCs has not been confirmed yet. Therefore, to further demonstrate whether PAK2 was a direct target of miR-455-3p and mediated the suppressive effect of miR-455-3p on the profibrotic phenotype, the two putative miR-455-3p target sites (wild-type (WT) or mutant (MUT) 3′ UTR target sequences) were cloned into a luciferase reporter vector. Luciferase activity of WT 3′ UTR of PAK2 was significantly inhibited in LX-2 cells transfected with miR-455-3p (Figure 3(b)); no change of luciferase activity was observed after the cotransfection of miR-455-3p with MUT 3′ UTR of PAK2 (Figure 3(b)). To further investigate whether miR-455-3p is able to successfully modulate PAK2 expression in HSCs, LX2 cells were transfected with miR-455-3p mimics or NC, we found that overexpression of miR-455-3p strongly attenuated the expression of PAK2 protein compared with the controls (Figures 3(c) and 3(d)). These findings suggested that miR-455-3p may repress the expression of PAK2 by directly targeting the 3′ UTR of PAK2.

### 3.4. HUC-MSCs Attenuate the Severity of Liver Fibrosis in Mice

**Figure 4:**
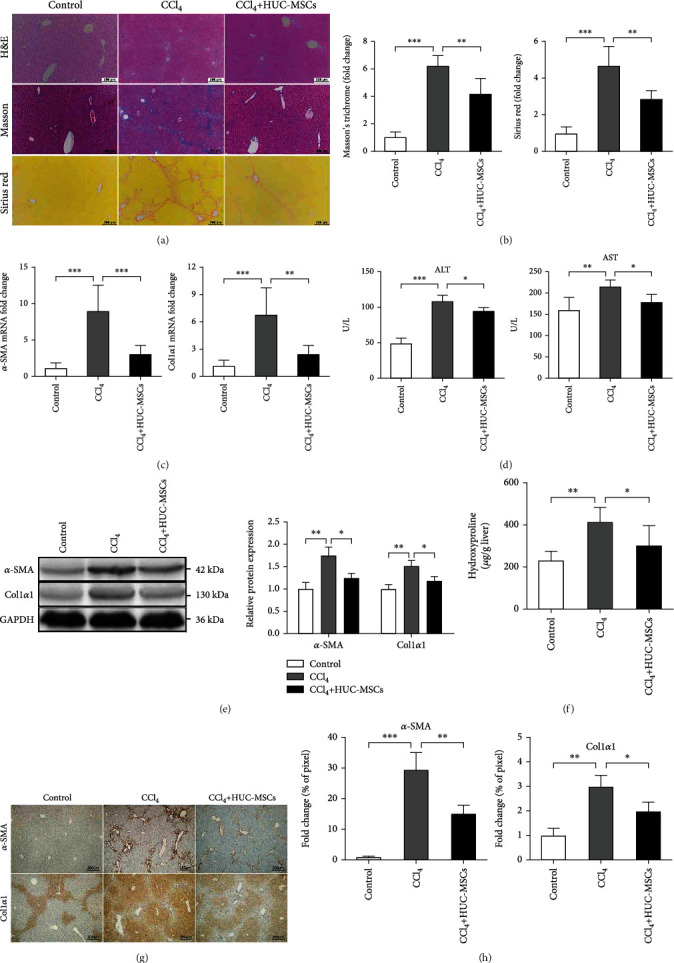
HUC-MSCs significantly attenuate severity of liver fibrosis in mice. (a) Representative images of H&E, Masson, and Sirius red staining of liver sections in olive oil-treated mice and mice of CCl_4_-induced liver fibrosis treated with PBS or HUC-MSCs. Scale bars, 200 *μ*m. (b) Relative fold change of Masson and Sirius red positive fibrosis areas compared with the olive oil-treated group. (c) qRT-PCR shows the expression of *α*-SMA and Col1*α*1 in the three groups. (d) Serum levels of ALT and AST were determined to indicate the extent of liver damage in the different groups. (e) The protein levels of *α*-SMA and Col1*α*1 in liver tissues were examined by Western blotting. (f) The amount of liver hydroxyproline was detected in the different groups. (g) Immunohistochemical analysis of mouse liver tissues for the expression of *α*-SMA and Col1*α*1. Scale bars, 200 *μ*m. (h) Quantification of immunohistochemical analysis. Percentages of immunoreactive (*α*-SMA and Col1*α*1) areas were expressed as relative values to those in oil-treated mouse livers. The quantitative data are represented as the mean ± SD. Each experiment was repeated three times. ^∗^*P* < 0.05, ^∗∗∗^*P* < 0.01, and ^∗∗∗^*P* < 0.001.

To further investigate the role of HUC-MSCs in hepatic fibrosis in vivo, HUC-MSCs were injected to mice once a week after 6 weeks of CCl_4_ administration during the 12 weeks of CCl_4_-induced fibrogenesis. At the end of the 12th week, we determined the degree of fibrosis in liver tissue by histology, Masson's trichrome, and Sirius red staining. We observed less liver damage and fewer fibrotic areas in the livers of mice injected with HUC-MSCs compared with PBS-treated controls (Figures 4(a) and 4(b)). Moreover, the mRNA and protein levels of profibrogenic markers, *α*-SMA and Col1*α*1 (Figures 4(c) and 4(e)), and the total collagen content (Figure 4(f)) was significantly downregulated in mice injected with HUC-MSCs. We also observed significantly lower *α*-SMA and Col1*α*1-positive areas (Figures 4(g) and 4(h)) in the livers of mice injected with HUC-MSCs, suggesting reduced fibrosis upon HUC-MSC inhibition. The levels of alanine aminotransferase (ALT) and aspartate aminotransferase (AST) in the serum of HUC-MSC infusion group were significantly downregulated compared with those of the PBS-treated group (CCl_4_ group) (Figure 4(d)), suggesting reduced liver injury upon HUC-MSC suppression in vivo. These results suggested that HUC-MSC injection could attenuate mouse liver fibrosis in this model.

### 3.5. HUC-MSCs Upregulating the Expression of miR-455-3p of CCl_4_-Induced Liver Fibrosis in Mice by Suppressing PAK2 Expression

**Figure 5:**
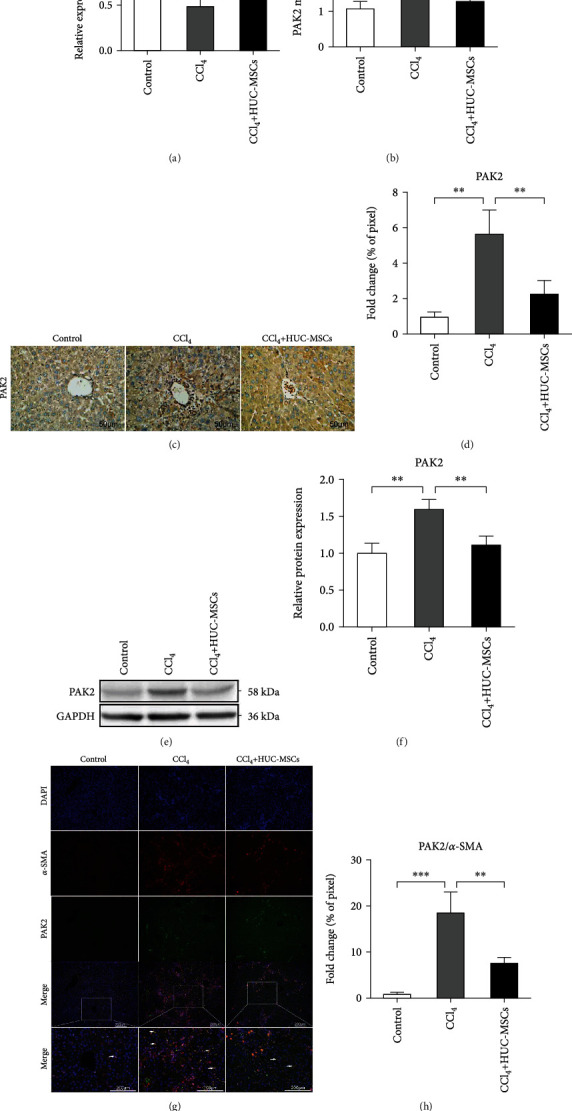
HUC-MSCs upregulate the expression of miR-455-3p of CCl_4_-induced liver fibrosis in mice by suppressing PAK2 expression. (a) The expression levels of miR-455-3p were examined in mouse hepatic tissues of the olive oil-treated group and CCl_4_-induced liver fibrosis groups treated with PBS or HUC-MSCs. (b) The expression levels of PAK2 mRNA in mouse hepatic tissues were examined by qRT-PCR. (c) Immunohistochemical staining of PAK2 in liver tissues of different groups. Scale bars, 50 *μ*m. (d) Quantification of PAK2-positive area in mouse hepatic tissues. (e) The protein levels of PAK2 in mice hepatic tissue were examined by Western blotting. (f) Quantitation of PAK2 protein levels from three independent Western blot analyses. (g) Immunofluorescence images of liver sections for the presence of DAPI (blue) or *α*-SMA (red) or PAK2 (green). The yellow areas indicated by the arrow are the coexpression regions of *α*-SMA and PAK2. Scale bars, 200 *μ*m. (h) Quantification of coexpression region of *α*-SMA and PAK2 in mouse hepatic tissues. The quantitative data are represented as the mean ± SD. Each experiment was repeated three times. ^∗^*P* < 0.05 and ^∗∗^*P* < 0.01.

To verify whether miR-455-3p is involved in the inhibitory effect of HUC-MSCs on liver fibrosis, we determined miR-455-3p levels in a mouse model of liver fibrosis, which was induced by injecting C57BL/6 mice with CCl_4_ for 12 weeks. The increased levels of miR-455-3p in fibrotic liver tissues of mice treated by HUC-MSCs compared with CCl_4_ group were confirmed by qRT-PCR analysis (Figure 5(a)). To examine the efficacy of miR-455-3p at the functional level, we analyzed the mRNA and protein level of PAK2, a target gene of miR-455-3p, which has been confirmed before. As shown in Figure 5(b), a significantly lower expression level of PAK2 was found in CCl_4_-treated mice injected with HUC-MSCs compared with PBS injection; we also found a significantly decreased PAK2 at protein level inspected by Western blotting and immunohistochemical analysis compared with the CCl_4_ group (Figures 5(c)–5(f)). To determine whether PAK2 was expressed in activated HSCs, we double-stained the liver tissue slices with *α*-SMA and PAK2 by immunofluorescence staining, and it could be seen that *α*-SMA and PAK2 were coexpressed in the space of Disse of the liver (the yellow areas indicated by the arrow) (Figure 5(g)), and the areas coexpression *α*-SMA and PAK2 in the HUC-MSC treatment group were significantly smaller than those in the CCl_4_ group (Figure 5(h)). It has been reported that PAK2 is highly expressed in activated HSCs [19], the immunohistochemical staining showed that PAK2 was positively expressed in hepatic portal area and sinuses of hepatic stromal cells (Figure 5(c), combined with immunofluorescence staining, and the results may indicate that PAK2 was positively expressed in activated HSCs in liver fibrosis. In conclusion, our findings revealed that HUC-MSCs alleviated hepatic fibrosis through upregulating miR-455-3p by targeting PAK2.

## 4. Discussion

Liver fibrosis is the end result of the most kinds of chronic liver damage and eventually will develop into liver cirrhosis, liver failure, and even liver cancer [20]. Activation of HSCs was believed to be the crucial component of this process [21]. However, there have been no effective therapeutic strategies for liver fibrosis up to now. Recently, MSCs have been shown to inhibit the activation of HSCs in vitro and improve liver fibrosis in animal models and in clinical trials [22–24]. However, the underlying mechanisms have not yet been fully elucidated.

Recent studies have focused on miRNA mechanisms in the pathophysiology of hepatic fibrosis and the activation of HSCs. miR-455-3p is generally regarded as an oncogene or tumor suppressor in different types of tumors [25, 26]. Recently, miR-455-3p has been demonstrated participating in fibrosis. For instance, upregulation of miR-455-3p could suppress idiopathic pulmonary fibrosis through repression of Bax expression [27]. miR-455-3p overexpression suppressed renal fibrosis through repressing Rho-associated protein kinase 2 (ROCK2) expression [28]. And miR-455-3p can inhibit liver fibrosis by alleviating HSC activation through suppressing heat shock transcription factor 1 (HSF1) expression [14]. A recent study showed that miR-455-3p derived from HUC-MSC exosomes has a promising treatment for acute liver injury [29]. However, it remains unclear whether miR-455-3p was involved in the mechanism of MSCs regulating liver fibrosis.

In the current study, we found that miR-455-3p was significantly upregulated in activated HSCs co-cultured with HUC-MSCs in vitro. In vivo, we found that miR-455-3p was upregulated in CCl_4_-induced hepatic fibrosis models treated by HUC-MSCs, which was in accordance with the results in vitro. Our findings suggested that miR-455-3p might play an important role in the MSC treatment of liver fibrogenesis, and its upregulation might be associated with the alleviation of hepatic fibrosis. However, how HUC-MSCs inhibit the activation of HSCs by regulating the expression of miR-455-3p is a question we need for a further study. According to the research by Shao et al. [29], IL-6 stimulation of HUC-MSCs can significantly upregulate the expression of miR-455-3p in its exosomes, while the exosomes with high expression of miR-455-3p can significantly reduce lipopolysaccharide- (LPS-) mediated liver injury in mice. We speculate that HUC-MSCs may inhibit the activation of HSCs and liver fibrosis by secreting exosomes rich in miR-455-3p.

To elucidate the target gene of miR-455-3p, we predicted it by bioinformatics analysis and confirmed that PAK2 was the target gene of miR-455-3p by luciferase reporter assay. PAK2 is one of the P21-activated kinases (PAKs) of the serine/threonine kinases family [30], which is a critical effector of the Rho family of small GTPases linked to cytoskeleton reorganization [31]. PAK2 is widely distributed in the whole body and regulates a variety of biological behaviors, including promoting tumorigenesis, cellular senescence, and organismal aging [32, 33]. TGF*β* has long emerged as a prominent master regulator of fibrogenesis [34]. The cellular responses evoked by TGF*β* were mediated by canonical SMAD signaling and noncanonical (non-SMAD) signaling, including mitogen-activated protein kinase (MAPK) and phosphoinositide 3-kinase (PI3K)/protein-serine-threonine kinase (Akt) signaling [35, 36]. As a downstream component of Akt, PAK2 has been reported as an activated receptor of TGF*β* in mesenchymal cells; PAK2 activation is essential for the proliferative/profibrotic action of TGF*β* [37]. PAK2 was also reported as a non-SMAD effector of TGF*β* in renal interstitial fibrogenesis; overexpression of PAK2 promotes renal fibrogenesis [38].

PAK2 was strongly expressed in activated HSCs [19], and it was highly likely that PAK2 positively modulates TGF*β*/Akt signaling in HSCs. In our study, we found that HUC-MSC treatment could inhibit the expression of profibrotic markers, upregulate miR-455-3p, and downregulate PAK2 in activated HSCs, and the effect could be enhanced by miR-455-3p mimics and attenuated by miR-455-3p inhibitors. In vivo, we also found that HUC-MSCs could upregulate miR-455-3p and inhibit PAK2 in a mouse hepatic fibrosis model. However, it has been reported that miR-455-3p can promote TGF*β*/SMAD signaling in chondrocytes and inhibit the development of osteoarthritis by directly targeting PAK2 [17]. Therefore, it might be that PAK2 positively modulates TGF*β*/Akt (non-SMAD) signaling in mesenchymal cells, while negatively regulating TGF*β*/SMAD signaling by attenuating the receptor-SMAD interaction in epithelial cells [39].

## 5. Conclusion

Our study demonstrated that HUC-MSCs significantly alleviate HSC activation and CCl_4_-induced liver fibrosis in a mouse model; overexpression of miR-455-3p by targeting PAK2 might be the underlying mechanism for the therapeutic effects of HUC-MSCs in hepatic fibrosis.

## Data Availability

The datasets generated and/or analyzed during the present study are available from the corresponding author upon reasonable request.

## Supplementary Materials

Supplementary Materials Supplementary Figure 1: HUC-MSCs inhibit HSC activation and promote miR-455-3p expression in a dose-dependent manner. (a) Comparison of mRNA levels of *α*-SMA measured by qRT-PCR of five groups with HUC-MSC : HSC ratios of 0 : 1, 1 : 25, 1 : 5, 1 : 1, and 5 : 1. (b) Comparison of mRNA levels of Col1*α*1 measured by qRT-PCR of the five groups. (c) Comparison of miR-455-3p levels measured by qRT-PCR of the five groups.
