# Supplementary material for: Human Umbilical Cord Mesenchymal Stem Cells Ameliorate Hepatic Stellate Cell Activation and Liver Fibrosis by Upregulating MicroRNA-455-3p through Suppression of p21-Activated Kinase-2
Source: Biomed Res Int. 2021 Feb 25;2021:6685605. doi: 10.1155/2021/6685605 (PMC7932777; doi:10.1155/2021/6685605)

**Figure Legends**

**Supplementary Figure 1** HUC-MSCs inhibit HSCs activation and promote miR-455-3p expression in a dose-dependent manner. (a) Comparison of mRNA levels of α-SMA measured by qRT-PCR of five groups with HUC-MSCs:HSCs ratios of 0:1, 1:25, 1:5, 1:1 and 5:1. (b) Comparison of mRNA levels of Col1α1 measured by qRT-PCR of the five groups. (c) Comparison of miR-455-3p levels measured by qRT-PCR of the five groups.


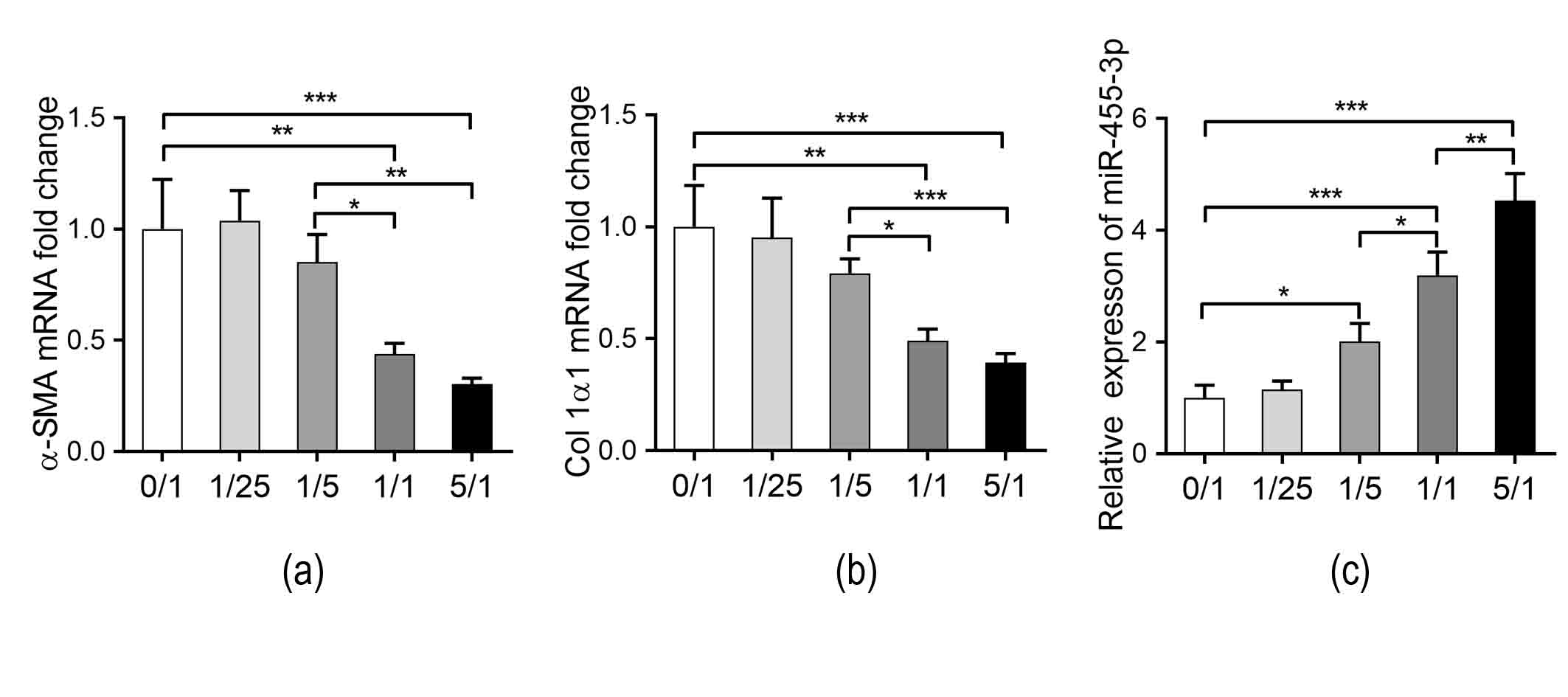

Supplement: Supplementary Materials — Supplementary Figure 1: HUC-MSCs inhibit HSC activation and promote miR-455-3p expression in a dose-dependent manner. (a) Comparison of mRNA levels of α-SMA measured by qRT-PCR of five groups with HUC-MSC : HSC ratios of 0 : 1, 1 : 25, 1 : 5, 1 : 1, and 5 : 1. (b) Comparison of mRNA levels of Col1α1 measured by qRT-PCR of the five groups. (c) Comparison of miR-455-3p levels measured by qRT-PCR of the five groups. [file 6685605.f1.docx]
